# A Case of Longitudinally Extensive Transverse Myelitis Following COVID-19 Infection

**DOI:** 10.7759/cureus.47604

**Published:** 2023-10-24

**Authors:** Jyotsna Gummadi, Meika Bhattachan, Athmananda Nanjundappa

**Affiliations:** 1 Department of Medicine, MedStar Franklin Square Medical Center, Baltimore, USA

**Keywords:** covid booster vaccine, urinary retention, dysphagia, back pain, acute transverse myelitis, letm, covid-19

## Abstract

One of the rare complications following acute COVID-19 infection is acute transverse myelitis (ATM). With only a few cases of ATM reported in the literature, an addition of longitudinally extensive transverse myelitis (LETM) diagnosed in our patient would underscore the complexity and diversity of neurological manifestations associated with this viral illness. A 54-year-old patient presented to the emergency department with fever, shortness of breath, nausea and vomiting. The patient’s nasopharyngeal swab for COVID-19 polymerase chain reaction (PCR) resulted positive. Few days later, the patient developed bilateral upper, lower extremities weakness, back pain, urinary retention and dysphagia. Subsequently, the clinical presentation, MRI, cerebrospinal fluid (CSF) and laboratory findings pointed toward LETM as a complication of COVID-19 infection over other differentials. The aggressiveness of this disease necessitated high-dose steroids and plasmapheresis, pain control medication and rehabilitation which led to a slight improvement in the neurological symptoms at the time of discharge to the rehabilitation facility.

## Introduction

As of August 27, 2023, globally over 770 million confirmed cases and over 6.9 million deaths have been reported due to COVID-19 [1]. Although the respiratory system complications of coronavirus disease 19 (COVID-19) have been the most frequent and life-threatening, a broad spectrum of neurological complications such as encephalopathy, stroke, Guillain-Barre syndrome, meningoencephalitis, acute necrotizing hemorrhagic encephalopathy, and inflammatory central nervous system syndromes have been reported in the literature [2]. Overall, the underlying causes of these complications are not well understood, and ongoing research is focused on exploring treatment possibilities. Here, we report a rare case of longitudinally extensive transverse myelitis following acute COVID-19 infection.

## Case presentation

A 54-year-old female with a past medical history (PMH) of fibromyalgia, migraine, and remotely diagnosed sarcoidosis presented to the emergency room (ER) with complaints of fever, left-sided chest pain, nausea, vomiting, chills, and runny nose that had been ongoing for a day. Chest pain was localized to the substernal, left chest regions, constant in nature, and associated with nausea, but not vomiting. She had received two doses of the COVID-19 vaccine but had not received a booster dose and denied exposure to sick individuals. Upon arrival at the ER, the patient was febrile with a temperature of 39.0°C, tachycardic with a heart rate of 134 bpm, and hypotensive with a blood pressure of 86/49 mm Hg. Physical examination did not reveal any pertinent findings. The patient received 3 liters of IV fluids, acetaminophen.

Laboratory tests, including the basal metabolic panel (BMP) and complete blood count, were within normal ranges (Appendix, Table 1). Lactic acid was within the normal range (<0.7 mmol/L). D-dimer was elevated, but CT angiography of the chest showed no evidence of pulmonary embolus or pneumonia. The patient tested positive for COVID-19 polymerase chain reaction (PCR). An electrocardiogram (EKG) showed a prolonged QTC of 534 ms.

The patient was admitted to the medical floor with a diagnosis of acute COVID-19 infection and managed at room air. Acute coronary syndrome was ruled out with serial undetectable troponin levels. On day three of hospitalization, the patient's condition deteriorated with worsening mental status and lactic acidosis (5.4 mmol/L) and was upgraded to the intermediate care unit (IMC). CT and MRI scans of the head showed no intracranial pathology. Liver function tests were unremarkable. She received IV normal saline for sepsis secondary to COVID-19, and lactic acidosis improved with IV fluids.

The patient developed urinary retention, requiring the placement of a Foley catheter on day five. On the same day, the patient reported weakness, especially in her lower extremities. Neurology was consulted, and a motor examination revealed diffusely absent tone, reflexes, and joint perception in both upper and lower extremities, as well as absent sensation in the distal aspect of bilateral (b/l) upper and lower extremities.

**Figure 1:**
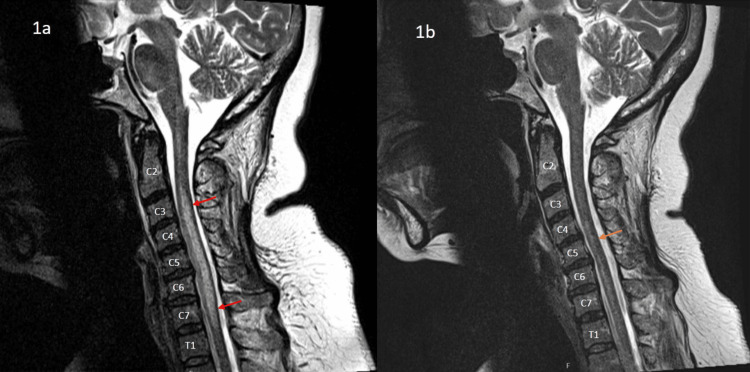
MRI scans 1a: Abnormal hyperintense T2 signal with mild fusiform swelling involving the cervical spinal cord from the level of C2-C3 down to the level of C7-T1 across the cross-section of the cord. This is associated with homogenous enhancement throughout the cross-section of the cord. 1b: Decreasing edema and T2 signal abnormality within the cervical spinal cord compared to the previous exam.

**Figure 2:**
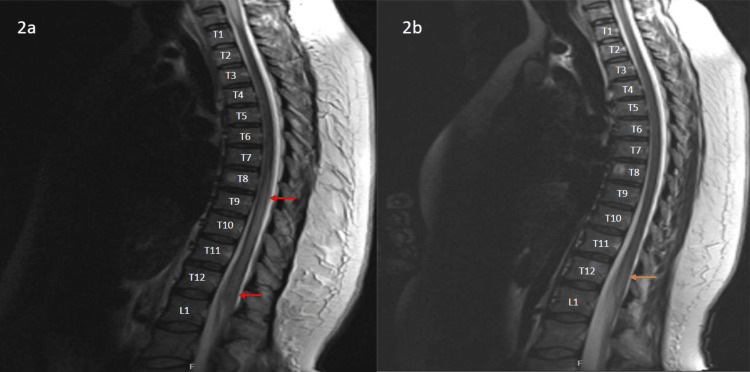
MRI scans 2a: Diffuse abnormal swelling and cross-sectional ill-defined enhancement throughout the mid to lower thoracic spinal cord from the level of T7-T8 to the conus medullaris. 2b: Significant improvement in the dilatation of the thoracic spine, now limited to the distal spinal cord at the level of T11 and T12 vertebral bodies. Faint enhancement of the conus is unchanged.

**Figure 3:**
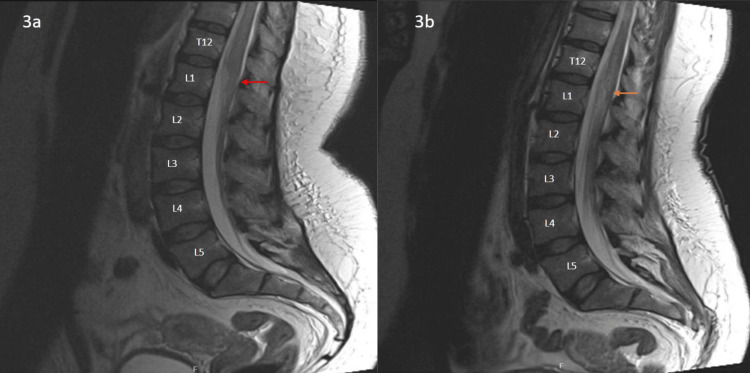
MRI scans 3a:  Diffuse swelling with increased T2 signal throughout the conus medullaris noted from at least the level of T11 down to the level of L1 with associated ill-defined enhancement. Conus terminates at L1. 3b: Significant improvement in the dilatation of the thoracic spine, now limited to the distal spinal cord at the level of T11 and T12 vertebral bodies. Faint enhancement of the conus is unchanged.

The patient was diagnosed with acute onset tetraplegia and longitudinally extensive transverse myelitis (LETM) from C2 to T1 and T8 to the conus. Following laboratory investigations were done to rule out the differentials including infectious diseases, autoimmune conditions, and vitamin deficiencies (Appendix, Table 2). Cerebrospinal fluid (CSF) analysis revealed an abnormal profile with elevated white blood cells, glucose, protein, and immunoglobulin G. On day five, MRI was done for cervical, thoracic, and lumbar regions of the spine without and with contrast and a lumbar puncture was done (Appendix, Figures 1a, 2a, 3a). The diagnosis for this complex case was COVID-19-associated longitudinally extensive transverse myelitis (LETM).

On day six of hospitalization, the patient received IV methylprednisolone 1 gm daily for a total of five days. Starting on day eight of hospitalization, the patient received plasmapheresis (albumin human 5% (DoT), 150 gm, 3,000 mL, 40 mL/kg, every other day, intravenous piggyback (IVPB)) on alternate days for a total of five sessions (stopped on day 16 of admission). During her hospital course, she also developed atrial fibrillation, abdominal distension, constipation, and ongoing back pain. Cryoprecipitate transfusions were administered for low fibrinogen levels (100 mg/dl) measured every 24 hours as long as the patient was on plasmapheresis. The patient also experienced the inability to move her right arm effectively to perform incentive spirometry, so she was monitored on end-tidal CO_2_ therapy for 72 hours. The patient complained of mild pain and pharyngeal dysphagia on day 17 for which speech-language pathology (SLP) therapy was consulted. Bedside swallowing evaluation was done, and the patient was recommended for diet downgrade to soft/bite-size solids with aspiration precautions, and 1:1 feeding assist due to bilateral upper extremity impairments.

On day 19 of hospitalization, MRI scans were repeated for the cervical, thoracic, and lumbar regions (Appendix, Figures 1b, 2b, 3b). Following the above treatment with steroids and plasmapheresis, a mild improvement in her motor function was noted in her left arm compared to her right arm. Unfortunately, there were no improvements in her lower extremities, resulting in a persistent inability to move her legs. She also complained of aching pain in both her arms and legs, as well as constant middle to low back pain. Despite these challenges, improvements were noted in her vision and swallowing function. The patient was also administered prophylactic meningococcal vaccine, for possible immunosuppressive treatment as outpatient.

Rheumatology was consulted given the complexity of her presentation. An extensive autoimmune workup was conducted (Table 2). Rheumatology suggested that the patient’s timeline of the symptoms was suggestive of viral transverse myelitis. The patient was discharged from the hospital to acute rehab on day 25 of hospitalization without any steroids.

## Discussion

Transverse myelitis (TM) includes a pathophysiologically heterogeneous syndrome characterized by acute or subacute spinal cord dysfunction resulting in paresis, sensory loss, and autonomic (bladder, bowel, and sexual) impairment below the level of the lesion. The most common potential etiologies can be broadly classified as para-infectious/post-infectious, toxin/drug-induced, paraneoplastic, autoimmune disorders, and acquired demyelinating diseases [3]. Transverse myelitis (TM) tends to occur most frequently in individuals during their second and fourth decades of life, often following a prior infection. Diagnosis typically involves using MRI to detect spinal cord abnormalities, and common treatments involve steroid therapy and plasmapheresis, with consideration given to avoiding any identified triggers [3].

A possible explanation for post- or para-infectious or post-vaccinal acute transverse myelitis (ATM) is that the immune response directed against the infectious agent may inadvertently target the central and peripheral nervous systems, potentially leading to neuronal damage or spinal tract injuries. Additionally, recent research indicating that severe acute respiratory syndrome coronavirus 2 (SARS-CoV-2) can enter human cells through angiotensin-converting enzyme 2 (ACE2) receptors, which are also found on the membranes of spinal cord neurons, raises the possibility of SARS-CoV-2 involvement in acute myelitis via ACE2 receptors [4].

In a review of 43 patients with COVID-19-associated acute transverse myelitis (ATM), 68% of cases exhibited a latency period of 10 days to six weeks after COVID-19 infection, possibly indicating a post-infectious neurological complication driven by the host's immune response, while 32% had a shorter latency of 15 hours to five days, suggesting a potential direct neurotropic effect of the SARS-CoV-2 virus on the nervous system [5]. Our report relates to the supporting literature as the patient experienced ATM-related symptoms five days after COVID-19 infection.

Magnetic resonance imaging is the diagnostic modality of choice for LETM. LETM includes a spinal cord lesion that extends over three or more vertebral segments on MRI. On axial sections, it typically involves the center of the cord over more than two-thirds of the spinal cord area [3]. This finding resonates with the MRI finding of cervical, thoracic, and lumbar regions in our patient which demonstrated diffuse abnormal swelling of the spinal cord from the level of C2 to T1 and T8 to conus.

The differential diagnoses for a developing myelopathy/ATM can be numerous, and identifying viral myelitis can pose a diagnostic challenge or, in certain instances, require ruling out other potential causes. Furthermore, the clinical characteristics, laboratory assessments (particularly, cerebrospinal fluid analysis), and imaging results offer valuable insights into neurological disorders [6]. Following a similar approach, we came up with various differentials including neuromyelitis optica spectrum disorder (NMOSD), myelin oligodendrocyte glycoprotein (MOG) LETM, LETM associated with sarcoidosis, viral myelitis, neoplasm, paraneoplastic disorders, systemic lupus erythematosus (SLE), Sjogren's syndrome, vitamin B12/Copper deficiency, thyroid disease, Lyme disease, neurosyphilis, and spinal cord infarct which were eventually ruled out (Appendix, Table 2).

Along with the diagnostic evaluation, we assumed a post-infectious etiology in terms of secondary immunogenic overreaction in our cases. Microorganisms, including HIV, *Treponema pallidum*, *Borrelia burgdorferi*, Lyme disease, *Cryptococcus neoformans*/*gattii*, Cytomegalovirus (CMV or human herpesvirus 5 (HHV-5)), herpes simplex virus, enterovirus, *Escherichia coli*, *Haemophilus influenzae*, human herpesvirus 6 (HHV-6), human parechovirus (HPeV), *Listeria monocytogenes*, *Neisseria meningitidis*, *Streptococcus agalactiae*, *Streptococcus pneumoniae, *and varicella-zoster virus (VZV) play a role in post-infectious acute myelitis and were investigated further. The result came negative for infectious origin. All the metabolic causes and medications were also ruled out as a cause of the ATM.

Another case study on LETMs presented with anatomically related symptoms but no observations of any inflammatory changes in CSF evaluation of which one case improved on steroids and the other on plasmapheresis revealing individual differences in COVID-19-mediated immunity [7]. Our study differs in that the patient had anatomically related symptoms and the CSF picture showed pleocytosis (>30/mm^3^), increased protein (85 mg/dl), and increased CSF IgG Index (0.80 ratio) showing an inflammatory pattern. Following the use of high-dose steroid and plasmapheresis, there was drastic clinical improvement in upper extremity movement and swallowing. However, the patient still complained of lower extremity pain, weakness, and continued back pain. The patient was discharged on pain medication, to the rehabilitation center with Foley’s catheter.

There have been some documented cases in medical literature of conditions including transverse myelitis and other neurological conditions occurring after COVID-19 infections of varying severity. Fortunately, most patients seem to achieve full neurological recovery [3]. However, our patient remained paraplegic following her hospital course.

The patient reported a remote medical history of sarcoidosis, which was initially diagnosed during her 20s with the patient recalling as a rash on her shoulder, not biopsy proven according to records review. Her sarcoidosis had been managed with inhalers and oral steroids, with no recurrence of symptoms since her 20s. Considering her medical history and the possibility of neuro-sarcoidosis, due to not being proven by biopsy in the past or during this current admission, the inability to rule out LETM associated with sarcoidosis is the limitation to our case report.

## Conclusions

In our case report, we present a case of longitudinally extensive transverse myelitis as a neurological complication of COVID-19. Here, no other causes of myelitis were identified after extensive workup. In conclusion, this report contributes supporting evidence for central nervous system (CNS) autoimmunity triggered by post-COVID-19 conditions, emphasizing the importance of considering COVID-19 as a potential cause of myelitis. Furthermore, the use of immunomodulatory treatments like steroids and plasmapheresis can lead to neurological improvement, as demonstrated by the patient described in this case.

## Appendices

**Table 1:** Significant laboratory findings comparing day 1, day 5, and other days of hospitalization CSF: cerebrospinal fluid, Syn: synthetic, Alb: albumin, hpf: high-power field.

	Parameters	Day 1	Day 5		Reference range
Biochemistry	Blood urea nitrogen	14 mg/dL	13 mg/dL		9-23 mg/dL
	Creatinine	0.84 mg/dL	0.68 mg/dL		0.50-0.80 mg/dL
	Creatine kinase	82 U/L	56 units/L		34-145 units/L
	Aspartate aminotransferase	15 U/L	62 units/L		0-33 units/L
	Bilirubin total	0.5 mg/dL	0.5 mg/dL		0.3-1.2 mg/dL
	Albumin	3.2 gm/dL	3.1 gm/dL		3.2-4.8 gm/dL
	Potassium	3.8 mmol/L	3.8 mmol/L		3.4-4.5 mmol/L
	Sodium	138 mmol/L	136 mmol/L		136-145 mmol/L
	Magnesium	1.8 mg/dL	2.1 mg/dL		1.6-2.6 mg/dL
	Chloride	102 mmol/L	106 mmol/L		98-107 mmol/L
	Calcium	8.6 mg/dL	7.9 mg/dL		8.7-10.4 mg/dL
	Bedside glucose	167 mg/dL	Not done		65-140 mg/dL
Hematology	White blood cell	7.78 k/µL	7.57k/µL		4-10.8 k/µL
		Lymphocyte=13.5%	Lymphocyte=55.2%		Lymphocyte=15%-45%
		Neutrophil=78.9%	Neutrophil=32%		Neutrophil=43%-75%
	Red blood cell	5.25 million/µL	10.4 million/µL		3.6-5 million/µL
	Hematocrit	15.1%	32.2%		34.5%-44%
	Platelets	292 k/µL	233 k/µL		145-400 k/µL
	Blood culture	Negative X1	Negative X 2		
Serology	C-reactive protein	Not done	10.4 mg/dL		0-10 mg/dL
Cerebral spinal fluid (CSF)		Not done	Tube 1	Tube 4	
	Appearance		Clear	Clear	
	Color		Colorless	Colorless	
	Post-spin CSF tube		Xanth absent	Xanth absent	
	RBC		113 /mm^3^	21/mm^3^	0-1/mm^3^
	WBC		31/mm^3^	38 /mm^3^	0-5/mm^3^
	Segs		68%	84 %	0%-7%
	Lymph		26%	13%	28%-96%
	Mono		6%	3%	16%-56%
	Glucose		51 mg/dl		40-70 mg/dl
	Protein		85 mg/dl		15-45 mg/dl
	Immunoglobulin G, CSF		11.5 mg/dl		0-6 mg/dl
	Albumin CSF		50 mg/dl		0-35 mg/dl
	IgG, Syn Rate, CSF		23.3 mg/day		<=8.0 mg/day
	CSF IgG Index		0.80 ratio		0.28-0.66 ratio
	CSF/serum Alb. index		19.8 ratio		0-9 ratio
	IgG/Alb ratio, CSF		0.23		0.09-0.25 ratio
	Oligoclonal bands number, CSF		Matching bands		0-1
	Oligo bands CSF		Negative		
Urine analysis (day 14)					
	Specific gravity		1.015		1.005-1.030
	Protein		Negative		Negative
	Glucose		Negative		Negative
	Blood		Large		Negative
	White blood cell		11-15/hpf		0-5/hpf
	Leukocyte esterase		Small		Negative
	Nitrite		Positive		Negative
Urine culture (day 25)	Negative				
Urine culture (day 26)	<10,000 cfu/ML mixed gram positive/negative organism. No further workup				

**Table 2:** Differential diagnoses and tests used to rule them out ANA: antinuclear antibodies, ds-DNA: double-stranded DNA, Sm: Smith antigen, SS-A/RO: Sjögren's syndrome A antigen/Ro antigen, SS-B/LA: Sjögren's syndrome B antigen/La antigen, ACE: angiotensin-converting enzyme, Cit: citrate, ACH: acetylcholine, CNS: central nervous system, MOG: myelin oligodendrocyte glycoprotein, AQP4: aquaporin-4, TSH: thyroid-stimulating hormone, FACS: fluorescence-activated cell sorting.

Categories	Causes		Ruled out methods
Para-infectious	Bacterial	Borrelia burgdorferi	Negative serum IgM and IgG
		Lyme disease Ab IgG	Negative
		*Escherichia coli* K1	Not detected
		Listeria monocytogenes	Not detected
		Neisseria meningitidis	Not detected
		Streptococcus agalactiae	Not detected
		Streptococcus pneumonia	Not detected
	Viral	HIV	Non-reactive
		Syphilis	Negative rapid plasma reagin
		Cytomegalovirus (CMV or HHV-5)	Not detected
		Herpes simplex virus ½	Not detected
		Enterovirus	Not detected
		Haemophilus influenzae	Not detected
		Human herpesvirus 6 (HHV6)	Not detected
		Human parechovirus (HPeV)	Not detected
		Varicella-zoster virus (VZV)	Not detected
Systemic inflammatory or autoimmune diseases	Systemic lupus erythematosus (SLE)		Negative ANA
			Negative serum anti-ds-DNA
			Negative anti-Sm antibodies
			Normal C3 complement level
	Sjogren’s syndrome		Negative serum anti-SS-A/RO
			Negative Anti-SS-B/LA
	Antiphospholipid syndrome		Negative serum antiphospholipid antibody
			Negative anti-cardiolipin antibody
			Negative beta 2 glycoprotein I Ab-IgM, IgG and IgA
	Neuro-sarcoidosis		Normal serum ACE
			Normal calcium level
	Systemic sclerosis		Negative serum anti-centromere antibody
			Negative anti-scl70
	Rheumatoid arthritis		Negative ANA
			Negative cyclic Cit peptide Ab IgG
	Sarcoidosis		Normal serum ACH
CNS autoimmune disorders	Multiple sclerosis (MS)		Normal CSF oligoclonal bands
			Normal serum anti-MOG antibodies
	Neuromyelitis optica (NMO)		Normal serum NMO-IgG (AQP4)
	Acute disseminated encephalomyelitis (ADEM) and cortical encephalitis		Normal serum anti-MOG antibodies
			Normal brain MRI
Trauma	Spinal cord compression (due to epidural abscess, tumor, or hematomas)		No evidence of any space-occupying or compressive condition in spinal cord MRI
Idiopathic vasculitis	Idiopathic CNS vasculitis		Negative anti-myeloperoxidase
	Wegener’s granulomatosis		Negative proteinase 3Ab
Metabolic and medication	Thyroid disorder		Normal TSH
	Vitamin D deficiency		Decreased (22.0 ng/ml)
	Vitamin B12 def		Normal
	Methylmalonic acid (MMA)		Normal
	Copper, plasma		Decreased
Genetic disorder	Paraneoplastic autoantibody evaluation of spinal fluid		Negative
	CNS demyelinating disease evaluation, serum		Negative NMO/AQP4 FACS (neuromyelitis optica/aquaporin 4 fluorescence-activated cell sorting (FACS)
			Negative myelin oligodendrocyte glycoprotein (MOG-IgG1) fluorescence-activated cell sorting (FACS)
